# Effects of Acute Fatigue on Balance Control of Alpine Skiing Athlete

**DOI:** 10.3390/life15050679

**Published:** 2025-04-22

**Authors:** Javier Riscart-López, Elena Jiménez-Herranz, Isabel Mendoza-Puente, Miguel Ángel Rosety-Rodríguez, Jorge Bastos-García, Manuel Rodríguez-Huguet, Juan José Ramos-Álvarez

**Affiliations:** 1 Department of Physical Education, Faculty of Education Sciences, University of Cádiz, 11519 Puerto Real, Spain; javier.riscart@uca.es (J.R.-L.); jorgen.bastosgarcia@gmail.com (J.B.-G.); 2 School of Sport Medicine, Faculty of Medicine, Complutense University of Madrid, 28040 Madrid, Spain; mariaelj@ucm.es (E.J.-H.); mendozapuenteisabel@gmail.com (I.M.-P.); jjramosa@ucm.es (J.J.R.-Á.); 3 Move-It Research Group, Biomedical Research and Innovation Institute of Cádiz (INiBICA), Puerta del Mar University Hospital, University of Cádiz, Plaza Fragela, s/n, 11003 Cádiz, Spain; 4 Independent Researcher, 11003 Cádiz, Spain; 5 Department of Nursing and Physiotherapy, University of Cádiz, 11009 Cádiz, Spain; manuel.rodriguez@uca.es

**Keywords:** ski, balance control, postural balance, fatigue, maximum effort

## Abstract

Background: Great physical requirements are necessary to maintain the entire body in a streamlined and aerodynamic position during downhill skiing. Balance control has an important role in alpine skiing and depends on muscle endurance and strength. The central processing of proprioception and the force capacity of muscle are altered by fatigue. The objective of this study was to assess the effects of fatigue and visual input on balance control in alpine skiing. Methods: Eleven male professional skiers participated in the study. Balance control with eyes open and eyes closed was assessed before and after performing a maximal effort specific alpine ski test. Variables: the total travel distance (TTD) (mm), radial area (RA) (mm^2^), ratio between TTD and RA (TTD/RA) (1/mm), mean center of pressure (COP) velocity (total length of the COP path per unit time) (mm/s), the mean mediolateral (ML) COP oscillation velocity (Lat_Vel) (mm/s), the mean anteroposterior (AP) COP oscillation velocity (AP_Vel) (mm/s), mean ML (MLD) (mm) and mean AP (APD) (mm) displacements of the COP and the distance from the ordinate origin (mean X and mean Y) (theoretical point where the COP should be) to the point at which the COP is located, and heart rate were measured. Results: The results showed differences in the variables related to postural control and balance before and after the stress test (*p* = 0.002–0.037). However, no differences were found when the results obtained with open and closed eyes were compared. Conclusions: The results showed that performance in alpine skiing could be negatively affected by fatigue. However, the dynamic parameters are not decreased by visual input during muscle fatigue.

## 1. Introduction

Alpine skiing has been one of the Winter Olympic sports since 1936. There are four main types of alpine skiing events (downhill, super giant slalom, giant slalom, and slalom), and these events are approximately 90 s to 2 min in duration. The skier has to accelerate as quickly as possible and to maintain correct form and technique from the starting gate to the remainder of the race [1]. Alpine skiing is an outdoor winter sport where balance control, weight transfer skills, isometric muscle strength in the lower extremities, mainly gravity, postural performance, coordination, and anaerobic–aerobic performance are required [2]. Among the variables, the effectiveness of the ride is largely determined by the balance [3,4].

During downhill skiing, static posture toward the skis is kept by the skier, as well as to maintain the balance of the entire “man-skis” relative to the ground, and its center of mass must project onto the base of support [4,5]. Modern alpine skiing techniques are characterized by a strong sense of lateral and forward–backward balance due to the wide angles of the inward lean of the body [3]. Therefore, great physical requirements are necessary to maintain the entire body in a streamlined and aerodynamic position or to make short radius turns during downhill skiing.

Static and dynamic balance in humans is regulated by a complex system of organs and mechanisms including the visual, vestibular, and somatosensory systems [6]. During ski turns, the share of vestibular information in posture control is increased by modifications in angular and linear acceleration; similarly, the afferent impulses from sight rise by the constant shift in space [6,7]. Therefore, skiing could be considered as a demanding sport because some studies have shown that attentional demands are greater for unstable than for stable balance conditions [8,9]. Sight plays a fundamental role in skiing, providing significant information related to body displacement kinematics, speed, and direction [10]. Research has shown a close relationship between dynamic balance and performance. Therefore, alpine ski training should be tailored to develop balance [10].

However, one variable that has not yet been explored in depth is the fatigue during downhill skiing. In this regard, balance control depends on muscle endurance and strength in developing postural control [11,12]. The central processing of proprioception and the force capacity of muscle are altered by fatigue, involving neural and muscular mechanisms [13,14,15]. This balance control largely depends on the proprioceptive input from the muscles of the legs and trunk [16]. It is known that proprioception is negatively affected by muscle fatigue. There has been some investigation into the role of proprioception in balance control, which has shown how increased local fatigue of the lower limbs decreases balance control [17,18,19]. Furthermore, balance control is more negatively affected by the fatigue of the proximal muscles of the lower limbs compared to distal muscles [18]. In alpine skiing, previous research has shown a high level of effort by knee extensor muscles during skiing [20,21]. Therefore, this should mean that performance should be seriously affected by the fatigue of the lower limbs.

Increasing fatigue is an important variable related to the balance control of an alpine skiing athlete, which can lead to lower performance. Unlike previous studies, the acute effects of fatigue on balance have not been studied previously in depth. Therefore, analysis enables researchers and coaches to align the actual performance used during training sessions with the scheduled load based on the level of fatigue based on balance. This makes the control of fatigue particularly useful to know the effects of the fatigue on physical performance. Although balance control plays an important role in alpine skiing, most studies have focused on physiology, biomechanics, and injuries. Few studies have evaluated the acute effects of fatigue on balance control [18,22,23]. Therefore, the main objective of the present study was to determine how balance control could be affected by muscle fatigue and visual input after performing a maximal effort alpine skiing test. Based on the previous literature, it was hypothesized that a high level of fatigue would lead to the decreased performance of alpine athletes.

## 2. Materials and Methods

### 2.1. Participants

**Figure 1:**
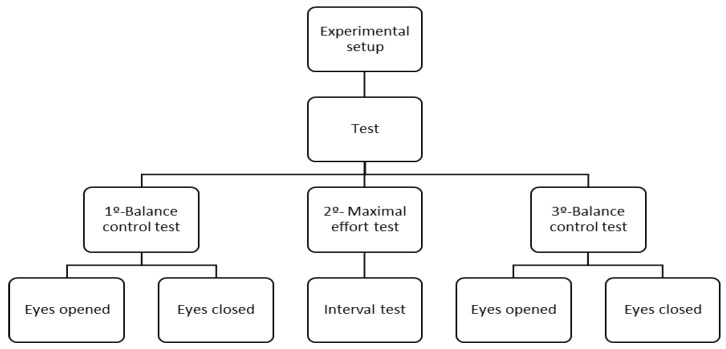
Flowchart of the experimental setup.

The study sample consisted of 11 male skiers (age: 23.8 ± 7.4 years; height: 177 ± 3.1 cm; body mass: 73.3 ± 2.3 kg) who were working as alpine ski instructors at the time the investigation was carried out. All subjects had uninterrupted experience practicing skiing for more than 10 years. The inclusion criteria of this sample were to ensure that skiers can perform the task accurately and to have a homogeneous sample. The participants had no physical limitations, health problems, or musculoskeletal injuries that could affect the evaluation. The measurements protocol performed are presented in Figure 1.

All participants were informed in a briefing about the purpose and methods of the study, as well as their right to withdraw at any time. All our procedures complied with the Helsinki Declaration, which outlines ethical principles for research involving humans. The study was approved by the CEIm Hospital Clínico San Carlos (identification code: 22/576-E) Ethics Committee in Madrid (Spain).

### 2.2. Materials

The Podoprint^®^ platform was used (Podoprint v2.6, Namrol Group, Barcelona, Spain) to asses postural sway [24,25]. The total travel distance (TTD) (mm), radial area (RA) (mm^2^), ratio between TTD and RA (TTD/RA) (1/mm), mean center of pressure (COP) velocity (total length of the COP path per unit time) (mm/s), the mean mediolateral (ML) COP oscillation velocity (Lat_Vel) (mm/s), the mean anteroposterior (AP) COP oscillation velocity (AP_Vel) (mm/s), mean ML (MLD) (mm), and mean AP (APD) (mm) displacements of the COP and the distance from the ordinate origin (mean X and mean Y) (theoretical point where the COP should be) to the point at which the COP is located were measured at a frequency of 100 Hz, using the manufacturer’s specific software [24,25]. The heart rate was recorded with a heart rate monitor (Polar Electro RS800cx, Kempele, Finland).

### 2.3. Balance Control Test

This was the first test that the subjects had to carry out. Before the measurements, the participants were informed about the entire process and the protocol for standing on the platform [26,27]. Participants were instructed to stand erect with their feet width the same as their shoulder width on the platform without shoes, motionless, and with their hands on their hips. They had to remain still in that position for 30 s while the measurements were made. Subsequently, a second measurement was taken in the same posture with eyes closed, within 30 s.

### 2.4. Maximal Effort Test

After completing the warm-up (5 min of jogging, 2 sets of 10 squats without additional load, 2 sets of 10 squats without additional load with ski, ski steps, several sets of progressively faster running accelerations with ski), participants performed an interval test, which consisted of a maximum-speed ski descent for 200 m (25% slope), with turning gates every 10 m. Upon reaching the end of the ski slope, they carried their skis on their shoulders and returned to the starting point. They had a maximum of 3 min to return to the starting point, put on their skis, and make a new descent. The test ended when two criteria were met: subjective exhaustion (modified 0–10 Borg scale (value 10 recorded)) and reaching at least 85% of the theoretical maximum heart rate [28,29]. Immediately, at the end of the interval-type test, the subjects returned to perform a second assessment of balance control with eyes open and closed, following the same protocol as in the initial assessment.

### 2.5. Statistical Analyses

Data are presented as the mean (M) ± standard deviation (SD). The normal distribution of data was confirmed using the Shapiro–Wilk’s test, and Leven’s test revealed the homogeneity of variance. A two-way repeated-measures ANOVA was used to analyze the effects of eye condition (open and closed) and time (pre-fatigue and post-fatigue) on the variables related to lengths, surface areas, velocities, and variations in the center of gravity. When a significant main effect was detected, pairwise comparisons were assessed using the Holm–Bonferroni test. Furthermore, ANOVA-RM effect sizes (ESs) were calculated using partial eta squared (η_p_^2^), with values <0.25, 0.26–0.63, and >0.63 considered small, medium, and large effect sizes, respectively [30,31]. On the other hand, in the pairwise comparisons, significance was assessed by calculating Cohen’s d ES [31]. Effect sizes were categorized as large (>0.8), moderate (0.5–0.8), small (0.2–0.5), and trivial (<0.2) [30,31]. Statistical significance was set at *p* < 0.05. All the statistical tests were performed using SPSS (version 18.0; SPSS, Chicago, IL, USA).

## 3. Results

Table 1 shows the values obtained for TTD, RA, and TTD/RA under pre- and post-fatigue conditions. TTDshowed statistically significant differences between pre- and post-fatigue conditions (86.8 ± 20.2 vs. 245.5 ± 38.5 mm; *p* = 0.002; η_p_^2^ = 0.633) for both open (*p* = 0.010; ES = 1.30) and closed eyes (*p* = 0.030; ES = 1.08). RA showed a trend towards statistical significance with higher values in the post-fatigue condition (149.4 ± 58.6 vs. 1319.5 ± 526.5 mm^2^; *p* = 0.054; η_p_^2^ = 0.322). The TTD/RA showed significant differences between the pre- and post-fatigue conditions, with a lower value in post-fatigue (1.54 ± 0.26 vs. 1.48 ± 0.26; 1/mm; *p* = 0.001; η_p_^2^ = 0.692). These significant differences were found for both open (*p* = 0.005; ES = 0.06) and closed eyes (*p* = 0.014; ES = 0.07). However, no significant differences were found between eye conditions or the interaction time and eye condition for TTD, RA, or TTD/RA (*p* > 0.05).

Table 2 shows the results of the variation of the dynamic parameters in the balance control test. For the velocity variables, no differences were observed between open and closed eyes for the interaction of time and eye condition (*p* > 0.05). A significant difference was found for the mean velocity with open eyes in the post-fatigue situation (*p* = 0.031; ES = 1.02), but no significant changes were observed for closed eyes in the post-fatigue situation (6.67 ± 1.31 vs. 2.78 ± 0.57 mm/s; *p* = 0.011; η_p_^2^ = 0.489). In Lat_Vel, higher values in the post-fatigue than the pre-fatigue condition were detected (4.70 ± 1.07 vs. 1.91 ± 0.42 mm/s; *p* = 0.003; η_p_^2^ = 0.399). A significant increase in AP_Vel in the post-fatigue condition was also detected (4.53 ± 0.86 vs. 1.91 ± 0.39 mm/s; *p* = 0.006; η_p_^2^ = 0.551), including these statistical differences with open eyes (*p* = 0.008; ES = 1.15) and a trend to statistical significance in closed eyes (*p* = 0.056; ES = 0.89).

Table 3 shows the results of the variation of the position of the center of mass. In relation with MLD, significant differences between the pre- and post-fatigue conditions were observed (1.69 ± 0.32 vs. 4.00 ± 0.98 mm; *p* = 0.037; η_p_^2^ = 0.368), with the statistical significances observed only with the open eye condition (*p* = 0.044; ES = 2.84), with no differences for eyes (*p* = 0.589) or the interaction time·eye (*p* = 0.892). On the other hand, in APD, differences were not observed for the factors time (*p* = 0.060), eye (*p* = 0.854), or the time·eye interaction (*p* = 0.423).

## 4. Discussion

The purpose of this study, which has been achieved, was to determine how balance control could be affected by muscle fatigue and visual input after performing a maximal effort alpine skiing test. The hypothesis planned was accepted, since it was a high level of fatigue that led to decreased performance regarding the balance of an alpine athlete.

In the present study, the postural sway assessment showed significant differences between pre-exercise and post-exercise with eyes open and eyes closed. These differences were observed both in dynamic and static parameters with eyes open and in dynamic parameters with eyes closed. Our results suggest that it could be considered that balance control could be affected by muscle fatigue.

Studies focused on changes in balance control with fatigue indicated that postural stability in either the sagittal or frontal planes have a greater deterioration when the proximal musculature of the lower limbs is fatigued [20]. Alpine skiing is characterized essentially by turns and this technical gesture, which demand greater sagittal balance where the proximal hip musculature is involved in the frontal plane and the knee extensor musculature in the sagittal plane [3]. This motion is a mix of static and dynamic muscle activity of the lower limbs, where high stress is induced on leg muscles and is a common cause of muscle fatigue [32,33]. Thus, during skiing, balance control could be negatively affected by lower limbs’ fatigue. Although balance control is an important variable in this sport, most of the studies carried out in alpine skiing have not focused on it [34]. It has been reported in the literature that the better the performance is, the greater the demand on the skier to have a greater mastery of sagittal balance ability and greater proprioceptive mechanism [35]. Thus, the study of balance could help determine the effectiveness of a specific exercise program on balance control in skiing and help minimize the negative effects of fatigue on it.

Our results have not shown significant differences when static parameters are compared between pre-exercise and post-exercise. It could be considered that the oscillations of the center of gravity were compensated by a correction in the visual inputs, and this would result in a compensatory motor response in the ankle and hip joint [36,37]. When the skier has his eyes closed, this visual stimulus is not produced. Therefore, there are no differences in the static parameters. However, in our case, it did not compensate for the oscillations in the center of gravity, since the dynamic parameters continued to provide significant differences. Research has shown that the visual system is the main source of afferent information, with a very important role in the control of motor skills [38]. Based on their visual feedback, the skier must make quick decisions and vary their speed, direction, or body position [39].

When comparing eyes open and eyes closed, it was observed that dynamic parameters have not shown significant differences. This could indicate that the post-exercise center of mass variations was not improved by visual stimulation. It is possible that other proprioceptive and vestibular factors were affected by fatigue after the test instead of visual afferences. To confirm this hypothesis, it would be necessary to carry out other studies in which there were a greater number of subjects, in different sports and in different situations of sensory conflict or vestibular stimulation.

Regarding the specific balance control training, previous studies have found that this kind of training allows the athlete to improve this capacity [40,41]. On the other hand, the practice of skiing has proven to be a suitable sports discipline for improving balance control in students [4,35], athletes [34], and people with disabilities [42] regardless of sex or skiing experience. In addition, other research has shown that low-level skiers had a greater balance control compared to high-level skiers, because the postural performance of high-level skiers is negatively affected by the repetitive wearing of ski boots [23]. Therefore, coaches and skiers should perform specific balance control training. However, the target population of this study was made up of experienced skiers, with a similar sporting level. These subjects presented similar pre-exercise stabilometric values, so the post-exercise loss of balance cannot be attributed to lack of experience. This could confirm the loss of balance related to maximum effort, with its consequences for performance in this sport. This information to monitor how postural control changes as a result of training can help them to better target the goal of their training. Workouts should focus on improving the endurance of the muscles involved in postural control, which could be evaluated during training and later applied to competition.

Different physical and physiological systems for posture control in alpine skiers can become the basis for future research on balance control. In this regard, analyzing the aerobic power (the maximum amount of energy your body can produce using oxygen), anaerobic power (the maximum amount of power you can exert during a short burst of intense activity), muscle strength (the maximum amount of force a muscle or muscle group can exert), and flexibility (the ability of a joint or series of joints to move through an unrestricted, pain free range of motion) could be a key point to understand the entire variables that could be affected on balance control [43]. However, further studies are needed to examine different effects on the balance control of single or a combination of physiological variables [21].

Regarding potential limitations. Firstly, regarding the number of participants analyzed, the study sample consisted of only 11 male skiers, which limits the generalizability of the findings to the broader alpine skiing population, including female athletes and skiers of different skill levels (i.e., top-level athletes). Further studies with larger samples are needed to confirm these results. However, the level of experience of the athletes in our sample was very similar, providing greater consistency to the results obtained. Secondly, no information was gathered about some proprioceptive and vestibular factors. It would have been interesting to analyze those variables; unfortunately, it was not possible to include such data in this study. Thus, the Sensory Organization Test (SOT) could be carried out to assess quantitatively an individual’s ability to use visual, proprioceptive, and vestibular cues to maintain postural stability in stance. Future studies comparing various types of static and dynamic exercises with eyes open and closed would be recommended to prospectively assess improvements in balance patterns.

## 5. Conclusions

The maximum exercise modifies the stabilometric parameters in skiers, with a greater oscillation of the center of gravity after it. Fatigue affects the skier’s postural control, so their performance could be reduced. Visual input does not improve the decrease in post-exertion dynamic parameters, but it does intervene in static parameters by increasing the average and maximum force exerted on the pressure platform.

## Figures and Tables

**Table 1:** The stabilometry and baropodometry platform values pre- and post-fatigue.

Variable	Eyes	Time		*p*-Value Time	η_p_^2^ Time	*p*-Value Eye	η_p_^2^ Eye	*p*-Value Eye·Time	η_p_^2^ Time·Eye
		Pre-Fatigue	Post-Fatigue						
TTD (mm)	Open	94.5 ± 83.0 *^A^	274.5 ± 188.3	0.002 *	0.633	0.386	0.076	0.607	0.027
	Closed	79.1 ± 55.8 *^B^	216.4 ± 179.3						
RA (mm^2^)	Open	187.3 ± 321.7	1725.8 ± 3139.0	0.054	0.322	0.467	0.054	0.545	0.038
	Closed	111.4 ± 84.7	913.2 ± 1952.3						
TTD/RA (1/mm)	Open	1.31 ± 0.94 *^A^	1.26 ± 0.91	0.001 *	0.692	0.192	0.164	0.412	0.068
	Closed	1.77 ± 1.05 *^B^	1.70 ± 1.08						

Data presented as M ± SD. TTD: Total travel distance; RA: radial area; TTD/RA: ratio between total travel distance and radial area; Significant differences for the time factor at *p* < 0.05. *: Significant difference between pre- and post-fatigue conditions; *^A^: Significant difference between pre- and post-fatigue with open eyes; *^B^: Significant difference between pre- and post-fatigue with closed eyes; TTD/RA: ratio between total travel distance and radial area.

**Table 2:** Values of the variation of the velocity of the center of mass in the balance control test.

Variable	Eyes	Time		*p*-Value Time	η_p_^2^ Time	*p*-Value Eye	η_p_^2^ Eye	*p*-Value Eye·Time	η_p_^2^ Time Eye
		Pre-Fatigue	Post-Fatigue						
Mean velocity (mm/s)	Open	2.93 ± 2.39 *^A^	7.55 ± 6.28	0.011 *	0.489	0.428	0.064	0.563	0.035
	Closed	2.65 ± 1.54	5.79 ± 5.60						
Lat_Vel (mm/s)	Open	2.07 ± 1.83	5.65 ± 5.71	0.028 *	0.399	0.325	0.097	0.471	0.053
	Closed	1.75 ± 1.08	3.71 ± 4.23						
AP_Vel (mm/s)	Open	2.01 ± 1.57 *^A^	4.84 ± 3.31	0.006 *	0.551	0.558	0.035	0.746	0.011
	Closed	1.81 ± 1.13	4.21 ± 3.83						

Data presented like M ± SD. Mean velocity: mean center of pressure (COP) velocity (total length of the COP path per unit time); Lat_Vel: the mean mediolateral (ML) COP oscillation velocity; the mean anteroposterior (AP) COP oscillation velocity; significant differences in factor time at *p* < 0.05. *: Significant difference between pre- and post-fatigue conditions; *^A^: significant difference between pre- and post-fatigue with open eyes.

**Table 3:** Values of the variation of the position of the center of mass in the balance control test.

Variable	Eyes	Time		*p*-Value Time	η_p_^2^ Eye	*p*-Value Eye	η_p_^2^ Time	*p*-Value Eye·Time	η_p_^2^ Eye·Time
		Pre-Fatigue	Post-Fatigue						
MLD (mm)	Open	1.92 ± 0.45 *^A^	4.37 ± 1.20	0.037 *	0.368	0.589	0.030	0.892	0.002
	Closed	1.45 ± 0.23	3.63 ± 1.59						
APD (mm)	Open	2.75 ± 1.95	4.12 ± 2.46	0.060	0.311	0.854	0.004	0.423	0.065
	Closed	2.21 ± 1.63	4.95 ± 5.06						

Data presented like M ± SD. MLD: mean mediolateral displacements of the COP and the distance from the ordinate origin (mean X and mean Y) (theoretical point where the COP should be) to the point at which the COP is located; APD: mean anteroposterior displacements of the COP and the distance from the ordinate origin (mean X and mean Y) (theoretical point where the COP should be) to the point at which the COP is located; significant differences in factor time at *p* < 0.05. *: Significant difference between pre- and post-fatigue conditions; *^A^: significant difference between pre- and post-fatigue with open eyes.

## Data Availability

The data showed in this study are available on request from the corresponding author. The data are not publicly available due to containing information that could compromise the privacy of research participants.

## References

[1] Müller E., Schwameder H. (2003). Biomechanical aspects of new techniques in alpine skiing and ski-jumping. J. Sports Sci..

[2] Klous M., Müller E., Schwameder H. (2014). Three-dimensional lower extremity joint loading in a carved ski and snowboard turn: A pilot study. Comput. Math. Methods Med..

[3] Staniszewski M., Zybko P., Wiszomirska I. (2016). Influence of a nine-day alpine ski training programme on the postural stability of people with different levels of skills. Biomed. Hum. Kinet..

[4] Jastrzębska A.D. (2020). Gender Differences in Postural Stability among 13-Year-Old Alpine Skiers. Int. J. Environ. Res. Public Health.

[5] Quatman-Yates C.C., Quatman C.E., Meszaros A.J., Paterno M.V., Hewett T.E. (2012). A systematic review of sensorimotor function during adolescence: A developmental stage of increased motor awkwardness?. Br. J. Sports Med..

[6] Cullen K.E. (2012). The vestibular system: Multimodal integration and encoding of self-motion for motor control. Trends Neurosci..

[7] Brauer S.G., Woollacott M., Shumway-Cook A. (2001). The interacting effects of cognitive demand and recovery of postural stability in balance-impaired elderly persons. J. Gerontol. A Biol. Sci. Med. Sci..

[8] Marsh A.P., Geel S.E. (2000). The effect of age on the attentional demands of postural control. Gait Posture.

[9] Gibson J.J. (1958). Visually controlled locomotion and visual orientation in animals. Br. J. Psychol..

[10] Raschner C., Hildebrandt C., Mohr J., Müller L. (2017). Sex Differences in Balance Among Alpine Ski Racers: Cross-Sectional Age Comparisons. Percept. Mot. Ski..

[11] Perrin P., Deviterne D., Hugel F., Perrot C. (2002). Judo, better than dance, develops sensorimotor adaptabilities involved in balance control. Gait Posture.

[12] Gandevia S.C. (2001). Spinal and supraspinal factors in human muscle fatigue. Physiol. Rev..

[13] Hunter S.K., Duchateau J., Enoka R.M. (2004). Muscle fatigue and the mechanisms of task failure. Exerc. Sport Sci. Rev..

[14] Sharpe M.H., Miles T.S. (1993). Position sense at the elbow after fatiguing contractions. Exp. Brain Res..

[15] Allum J.H., Bloem B.R., Carpenter M.G., Hulliger M., Hadders-Algra M. (1998). Proprioceptive control of posture: A review of new concepts. Gait Posture.

[16] Forestier N., Teasdale N., Nougier V. (2002). Alteration of the position sense at the ankle induced by muscular fatigue in humans. Med. Sci. Sports Exerc..

[17] Salavati M., Moghadam M., Ebrahimi I., Arab A.M. (2007). Changes in postural stability with fatigue of lower extremity frontal and sagittal plane movers. Gait Posture.

[18] Chollet M., Hintzy F., Cross M.R., Delhaye C., Morel B., Monjo F., Samozino P. (2024). Fatigue-induced alterations in force production, trajectory and performance in alpine skiing. J. Sports Sci..

[19] Berg H.E., Eiken O., Tesch P.A. (1995). Involvement of eccentric muscle actions in giant slalom racing. Med. Sci. Sports Exerc..

[20] Clarys J.P., Alewaeters K., Zinzen E. (2001). The influence of geographic variations on the muscular activity in selected sports movements. J. Electromyogr. Kinesiol..

[21] Neumayr G., Hoertnagl H., Pfister R., Koller A., Eibl G., Raas E. (2003). Physical and physiological factors associated with success in professional alpine skiing. Int. J. Sports Med..

[22] Noé F., Paillard T. (2005). Is postural control affected by expertise in alpine skiing?. Br. J. Sports Med..

[23] Azevedo N., Ribeiro J.C., Machado L. (2022). Balance and Posture in Children and Adolescents: A Cross-Sectional Study. Sensors.

[24] Massó-Ortigosa N., Rey-Abella F., Gutiérrez-Vilahú L., Milà R., Guerra-Balic M., Oviedo G.R. (2024). Analysis of the centre of pressure in bipedal stance among individuals with and without intellectual disabilities, individuals with Down syndrome and dancers with Down syndrome. J. Intellect. Disabil. Res..

[25] Oviedo G.R., Guerra-Balic M., Baynard T., Javierre C. (2014). Effects of aerobic, resistance and balance training in adults with intellectual disabilities. Res. Dev. Disabil..

[26] Pascual-Vaca A.O., Punzano-Rodríguez R., Escribá-Astaburuaga P., Fernández-Domínguez J.C., Ricard F., Franco-Sierra M.A., Rodríguez-Blanco C. (2017). Short-Term Changes in Algometry, Inclinometry, Stabilometry, and Urinary pH Analysis After a Thoracolumbar Junction Manipulation in Patients with Kidney Stones. J. Altern. Complement. Med..

[27] Yoon J.J., Yoon T.S., Shin B.M., Na E.H. (2012). Factors affecting test results and standardized method in quiet standing balance evaluation. Ann. Rehabil. Med..

[28] Tanaka H., Monahan K.D., Seals D.R. (2001). Age-predicted maximal heart rate revisited. J. Am. Coll. Cardiol..

[29] Midgley A.W., McNaughton L.R., Polman R., Marchant D. (2007). Criteria for determination of maximal oxygen uptake: A brief critique and recommendations for future research. Sports Med..

[30] Lakens D. (2013). Calculating and reporting effect sizes to facilitate cumulative science: A practical primer for t-tests and ANOVAs. Front. Psychol..

[31] Ferguson C.J. (2009). An effect size primer: A guide for clinicians and researchers. Prof. Psychol. Res. Pract..

[32] Kröll J., Wakeling J.M., Seifert J.G., Müller E. (2010). Quadriceps Muscle Function during Recreational Alpine Skiing. Med. Sci. Sports Exerc..

[33] Müller E., Gimpl M., Kirchner S., Kröll J., Jahnel R., Niebauer J., Niederseer D., Scheiber P. (2011). Salzburg Skiing for the Elderly Study: Influence of alpine skiing on aerobic capacity, strength, power, and balance. Scand. J. Med. Sci. Sports.

[34] Bang D.H., Shin W.S. (2017). Effects of an intensive Nordic walking intervention on the balance function and walking ability of individuals with Parkinson′s disease: A randomized controlled pilot trial. Aging Clin. Exp. Res..

[35] Wojtyczek B., Pasławska M., Raschner C. (2014). Changes in the balance performance of polish recreational skiers after seven days of alpine skiing. J. Hum. Kinet..

[36] Herdman S.J. (1989). Exercise strategies for vestibular disorders. Ear Nose Throat J..

[37] Herman K., Barton C., Malliaras P., Morrissey D. (2012). The effectiveness of neuromuscular warm-up strategies, that require no additional equipment, for preventing lower limb injuries during sports participation: A systematic review. BMC Med..

[38] Abahnini K., Proteau L., Temprado J.J. (1997). Evidence Supporting the Importance of Peripheral Visual Information for the Directional Control of Aiming Movement. J. Mot. Behav..

[39] Decroix M., Wazir M.R.W.N., Zeuwts L., Deconinck F.F.J.A., Lenoir M., Vansteenkiste P. (2017). Expert—Non-expert differences in visual behaviour during alpine slalom skiing. Hum. Mov. Sci..

[40] Stanton R., Reaburn P.R., Humphries B. (2004). The effect of short-term Swiss ball training on core stability and running economy. J. Strength. Cond. Res..

[41] Yaggie J.A., Campbell B.M. (2006). Effects of balance training on selected skills. J. Strength Cond. Res..

[42] Nasuti G., Temple V.A. (2010). The risks and benefits of snow sports for people with disabilities: A review of the literature. Int. J. Rehabil. Res..

[43] Turnbull J.R., Kilding A.E., Keogh J.W. (2009). Physiology of alpine skiing. Scand. J. Med. Sci. Sports.

